# Moxibustion Regulates Amino Acid Metabolism Pathways to Improve Cartilage Damage in Knee Osteoarthritis Model Rats

**DOI:** 10.7759/cureus.97527

**Published:** 2025-11-22

**Authors:** Liu Lei, Wei Xia, Gao Zi Zhan, Duan Wen Xiu, Yu Qing, Wang Jie, Cai Rong Lin, Wu Zi Jian

**Affiliations:** 1 School of Acupuncture and Moxibustion and Massage, Anhui University of Chinese Medicine, Hefei, CHN; 2 Integrative/Complementary Medicine, Huaibei People's Hospital, Huaibei, CHN; 3 College of Traditional Chinese Medicine, Hubei College of Chinese Medicine, Jingzhou, CHN; 4 Acupuncture and Moxibustion Meridian Research Institute, Anhui Academy of Chinese Medicine, Hefei, CHN

**Keywords:** amino acid metabolism, cartilage injury, chinese medicine, knee osteoarthritis, moxibustion

## Abstract

Objective: Knee osteoarthritis (KOA) is a chronic joint condition characterized by progressive cartilage degeneration and bone remodeling. Conventional drug treatments often provide limited relief and may cause adverse effects. As a nonpharmacological therapy in traditional Chinese medicine, moxibustion has been demonstrated to alleviate KOA-related pain, reduce inflammation, and ameliorate cartilage damage through immunomodulatory and metabolic pathways. This study aimed to explore the involvement of amino acid metabolism in the mechanism of the protective effects of moxibustion on cartilage injury in a rat model of KOA.

Methods: Sprague-Dawley rats were randomly assigned to three groups: normal, KOA model, and moxibustion treatment. The KOA model was established by intra-articular injection of 0.05 mL of a 30 mg/mL sodium iodoacetate solution into the right knee joint cavity of model and moxibustion groups. The moxibustion group received mild moxibustion treatment at Stomach Meridian 36 (ST36) on the right hind limb for 30 min daily over 30 consecutive days. The mechanical pain threshold was measured using Von Frey filaments, while joint swelling was assessed with an electronic caliper. Hematoxylin and eosin staining was performed to evaluate cartilage morphology, and a transmission electron microscope was used to observe chondrocyte ultrastructure. Levels of matrix metalloproteinase-13 (MMP-13) and interleukin-1β (IL-1β) in synovial tissue were quantified using enzyme-linked immunosorbent assay. Alterations in cartilage amino acid metabolites were analyzed using liquid chromatography-mass spectrometry.

Results: In the normal group, the cartilage exhibited a smooth surface, with an intact matrix structure, and normal chondrocyte morphology. In contrast, the KOA model group presented with cartilage surface roughness, inflammatory cell infiltration, fibrosis, reduced chondrocyte density, nuclear pyknosis, and organelle damage after 30 days of chemical induction. Moxibustion alleviated structural damage, preserving hyaline cartilage, reducing inflammation and fibrosis, and improving chondrocyte morphology. Model rats displayed significant knee swelling (*P *< 0.0001), decreased pain thresholds (​​​​​​​*P *< 0.0001), and increased MMP-13 (*P *< 0.0001) and IL-1β expression (*P *< 0.0001). After moxibustion treatment, the pain threshold significantly increased (​​​​​​​*P *= 0.045), swelling was reduced (​​​​​​​*P *< 0.0001), and MMP-13 (​​​​​​​*P *= 0.0004) and IL-1β (​​​​​​​*P *= 0.002) levels significantly decreased. Moxibustion also downregulated the alanine and glutamine levels in cartilage (*P *< 0.0001). The Kyoto Encyclopedia of Genes and Genomes pathway analysis revealed that alanine, aspartate, and glutamate metabolic pathways were the most significantly affected.

Conclusion: Moxibustion at ST36 can significantly improve joint swelling, pain, synovitis, and chondrocyte injury in rats with KOA. It modulates immune response and cartilage synthesis and degradation through alanine, aspartate, and glutamate metabolic pathways, thereby exerting anti-inflammatory, analgesic, and protective effects on cartilage injury.

## Introduction

Knee osteoarthritis (KOA) is a chronic joint disorder characterized by progressive degeneration of articular cartilage and compensatory bone growth. Its clinical manifestations include knee pain, swelling, and limited joint mobility. The inflammatory process originates in the synovium, activating the immune system and engaging both humoral and cellular inflammatory mediators. Pathological changes involve structural damage to the articular cartilage, infrapatellar fat pad, meniscus, synovium, and surrounding ligaments [1,2]. Although nonsteroidal anti-inflammatory drugs and opioid analgesics are commonly used in the clinical treatment of KOA, these therapies often provide limited long-term efficacy and are associated with significant adverse effects. Therefore, developing new, safe, and effective therapeutic strategies is urgently needed.

Traditional Chinese medicine (TCM)-based nonpharmacologic therapies have demonstrated notable efficacy in alleviating pain and improving the quality of life of patients with KOA, attracting increasing attention in recent years [3]. Among them, moxibustion, one of the characteristic TCM treatments, is widely used to treat arthritis. It is a simple, noninvasive, and effective method that can alleviate joint pain and swelling, protect articular cartilage, suppress inflammation, and improve the quality of life of the patient [4,5].

Previous research demonstrated that moxibustion significantly decreases inflammatory cytokine levels, alleviates knee pain and swelling in KOA model rats, and reduces cartilage destruction, highlighting its anti-inflammatory and immunomodulatory properties [6,7]. KOA-induced articular cartilage degeneration is closely associated with metabolic dysregulation, including glucose, lipid, and amino acid metabolism within local tissues and cells [8]. Because metabolic homeostasis is essential for the maintenance of cartilage function and self-repair, disturbances in these pathways may exacerbate joint injury. Amino acid metabolism is crucial for both the synthesis and degradation of the cartilage matrix. This study aimed to investigate the effects of moxibustion at ST36 on knee swelling, the mechanical pain threshold, the chondrocyte ultrastructure, and matrix metalloproteinase-13 (MMP-13) and interleukin-1β (IL-1β) levels in the synovial tissue of sodium iodoacetate-induced KOA rats. Additionally, we analyzed amino acid metabolite changes in rat cartilage using liquid chromatography-mass spectrometry (LC-MS)-based targeted metabolomics to explore, preliminarily, the mechanism by which moxibustion ameliorates KOA cartilage injury.

## Materials and methods

Animals

The animal sample size for this experiment was determined according to previous literature reports [9]. A total of 30 healthy male Sprague-Dawley rats aged 12 weeks and weighing 270 ± 30 g were obtained from the Shandong Experimental Animal Center (animal use license Number: SCXK (Lu) 2019-0003). The rats were randomly allocated into three groups, namely, the normal, model, and moxibustion treatment groups, with each group comprising 10 rats. The rats were housed under controlled environmental conditions: temperature of 27 ± 0.5 ℃, relative humidity 55 ± 5%, and a 12-hour light/dark cycle, with unrestricted access to food and water. All animal experiments in this study were supervised and reviewed by the animal ethics committee of Anhui University of Chinese Medicine (Animal ethics Number: AHUCM-rates-2023040).

Induction of KOA

A week after adaptive feeding, the rats in both the model and moxibustion groups were anesthetized with 4% isoflurane inhalation. The right knee joint and surrounding area were shaved and disinfected with 75% alcohol. The operator flexed the right knee of the rat with the left hand injected sodium iodoacetate solution into the joint cavity through the lower lateral margin of the patella to induce the KOA model. Normal rats received no treatment. According to previously published methods [10], each rat was injected with 50 μL of 30 mg/mL sodium iodoacetate solution. The model was allowed to develop for seven days, after which the obvious swelling of the right knee joint confirmed successful induction of the KOA model.

Animal intervention

According to the Acupoint Atlas of rats [11], the ST36 acupoint is located posterolateral to the knee joint, approximately 3 mm below the fibular capitulum. In the moxibustion group, the right ST36 point was selected for treatment. Based on previous studies from our group [12], mild moxibustion was performed using a 0.9 cm diameter moxa stick, kept at a 2 cm distance from the skin for 30 min per session, daily for 30 consecutive days. The normal and model groups did not receive moxibustion. Rats that died during the experimental period were excluded, leaving eight rats per group for the final statistical analysis.

Measurement of joint swelling

An electronic Vernier caliper (DL90150B type; Deli, Ningbo, China) was used to measure and record the diameter of the right knee joint in each group before and after modeling, and after moxibustion treatment as an index of joint swelling. The white ligament width of the knee joint was used as the measurement reference. Joint diameters were recorded in centimeters (cm) for all groups.

Measurement of mechanical pain threshold

After completing joint swelling assessments, the mechanical pain threshold for each rat was determined using von Frey hairs (Aesthesio; Danmic Global, San Jose, CA, USA). The central plantar surface of the right hand paw was gently stimulated with von Frey filaments until the rat withdrew, lifted, or licked the paw, which was recorded as a positive response. The interval between each stimulus was at least 30 seconds. If no response occurred, the next higher filament was applied; if a positive response was observed, the next lower filament was used. This process continued until a consistent withdrawal threshold was established. The final pressure value was expressed in grams (g).

General view of the cartilage surface

After joint swelling measurement, the rats were euthanized under anesthesia, the knee joint capsule was exposed, and the patella was excised. The femoral condyle and tibial plateau cartilage surfaces were observed for color, texture, and morphological changes, which were photographically documented.

Hematoxylin-eosin (HE) staining observation

After euthanasia, the femoral condyle of the right knee was excised and fixed in 4% paraformaldehyde for 24 hours. The sample was decalcified using a 10% EDTA solution (G1105 slow type; Servicebio, Wuhan, China). The tissues were then dehydrated through an ethanol gradient, followed by clearing with a 1:1 mixture of absolute ethanol and xylene and then with xylene alone. Paraffin embedding was performed after two rounds of wax infiltration. The paraffin sections were dewaxed with xylene, rehydrated in an ethanol gradient, and rinsed with distilled water. Hematoxylin staining was followed by acid alcohol differentiation (1% hydrochloric acid ethanol), bluing with phosphate-buffered saline (PBS), and counterstaining with 0.5% eosin in ethanol. After being washed with 95% ethanol and immersed in 100% ethanol for five minutes, the sections were cleared with xylene and mounted using neutral resin. The morphology of the cartilage tissue was observed and photographed under a light microscope.

Transmission electron microscopy (TEM) observation

Cartilage tissue (approximately 0.5 cm³) from the knee of the rat was cut and immediately fixed in 2.5% glutaraldehyde for 24 hours. After fixation, samples were rinsed in PBS for six hours and subsequently fixed with 1% osmium acid for two hours. Graded dehydration was performed using ethanol solutions, followed by a 30-minute immersion in propylene oxide, and then embedded in epoxy resin. After the block was dried and trimmed in an oven, an ultrathin microtome (UC-7; Leica, Teaneck, NJ, USA) was used to cut ultrathin 70 nm thick slices. The slices were then collected on copper grids and stained with lead and uranium. The ultrastructures of the chondrocytes were examined using transmission electron microscopy (JEM1400; JEOL, Tokyo, Japan), with images captured using a digital camera (Morada G3; Munters, Hamburg, Germany).

Enzyme-linked immunosorbent assay (ELISA) detection

Synovial tissue from rat knee joints was carefully dissected, rinsed with precooled PBS to remove blood and debris, snap-frozen with liquid nitrogen, and stored at -80 ℃ until analysis. Before testing, the tissue was weighed, minced, and homogenized in PBS (1:9 w/v) using a glass homogenizer on ice. The homogenate was then subjected to ultrasonic disruption to further lyse the tissue cells. The supernatant was collected for analysis after centrifugation of the homogenate. The detection steps were performed according to the instructions of the rat MMP-13 ELISA kit and rat IL-1β ELISA kit (Jonlnbio, Shanghai, China).

LC‒MS-targeted metabonomics detection

Cartilage tissue was extracted from the knee joints of the rats; the samples were rinsed with precooled PBS to remove blood and impurities. Approximately 50 mg of each sample was accurately weighed into a 2 mL centrifuge tube. The samples were extracted with 600 μL of a 10% formic acid solution in a 1:1 methanol: water mixture. The mixture was vortexed for 30 seconds, then ground in a tissue grinder at 55 Hz for 90 seconds, and the supernatant was taken after centrifugation. The supernatant was diluted 50-fold with 10% formic acid in methanol: water (1:1 w/v) and vortexed for 30 seconds. Next, 100 μL of Trp-d3 (10 ng/mL) was added to 100 μL of the diluted supernatant, and the mixture was vortexed again for 30 seconds. The mixture was filtered through a 0.22 μm filter and transferred to an LC-MS vial for analysis.

Chromatographic separation was performed using a ZORBAX Eclipse XDB-C18 column (4.6 × 150 mm; Agilent, Santa Clara, CA, USA) for liquid chromatography. The injection volume was 5 μL, and the column temperature was 40 ℃. The mobile phase consisted of (A) 10% methanol in water with 0.1% formic acid and (B) 50% methanol in water with 0.1% formic acid. Mass spectrometry was performed using an electrospray ionization source in positive ionization mode. The ion source was set to 500 ℃, and the ion spray voltage was 5500 V. The collision and curtain gas pressures were maintained at 6 psi and 30 psi, respectively, while both nebulizer and auxiliary gas pressures were maintained at 50 psi. The multiple reaction monitoring mode was used for data acquisition. Chromatographic peak areas and retention time were extracted using MultiQuant software. Retention times were corrected using amino acids and derivative standards, and the metabolites were identified accordingly.

Statistical analyses

Statistical analysis and graph generation were performed using GraphPad Prism software (version 7.0; La Jolla, CA, USA). Data were first assessed for normality. Normally distributed data are presented as the means ± standard deviations. One-way analysis of variance was used for comparisons among groups. Pairwise comparisons were conducted using the Tukey test for equal variances and the Dunn test for unequal variances. Statistical significance was determined at P < 0.05. Metabonomic data were analyzed using the BioDeep Big Data Cloud Intelligent Analysis Platform (https://www.biodeep.cn). The R language was used to analyze and plot the data. One-dimensional statistical analyses included t-tests and fold change (FC) analysis.

## Results

**Figure 1:**
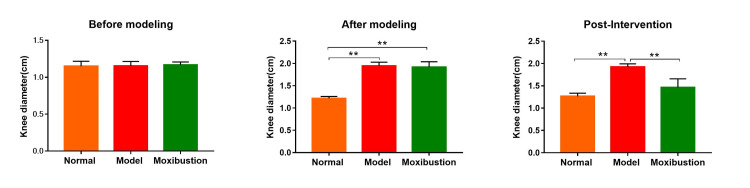
Comparison of the degree of joint swelling in each group. n=8 per group. Data are expressed as mean ± SD and analyzed using one-way ANOVA followed by Tukey's test. Significance levels are indicated as follows: ** for *P*<0.0001.

Before modeling, the bilateral knee joints of all were normal, with no visible morphology differences. Compared with that in the normal group, joint swelling in moxibustion and model groups was significantly greater (P < 0.0001). Compared with the model group, the moxibustion group exhibited a significant reduction in joint swelling (P < 0.0001) (Figure 1).

**Figure 2:**
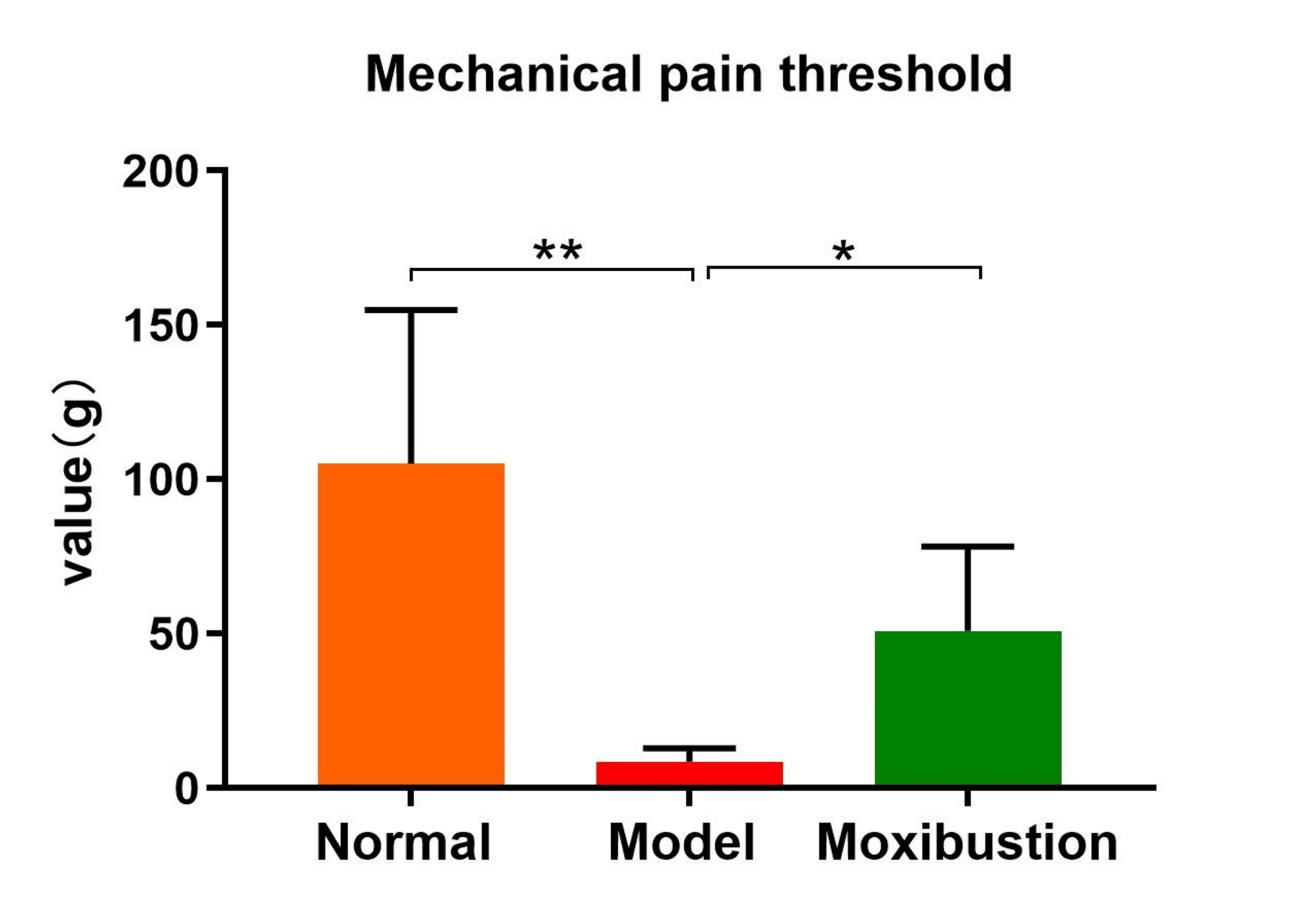
Comparison of mechanical pain thresholds in each group. n=8 per group. Data are expressed as mean ± SD and analyzed using one-way ANOVA followed by Tukey's test. Significance levels are indicated as follows: * for *P*<0.05 and ** for *P*<0.0001.

Compared with the normal group, the model group presented a significantly lower mechanical pain threshold (P < 0.0001). Compared with the model group, the moxibustion group presented a considerably greater mechanical pain threshold post-intervention (P < 0.05) (Figure 2).

In the normal group, the articular cartilage surfaces of the femoral condyle and tibial plateau were smooth, translucent, shiny, and pink, with no visible damage. In the model group, the femoral condyle and tibial plateau exhibited a rough articular cartilage surface lacking luster, with evident inflammatory hyperplasia, fibrosis, and severe erosion of the hyaline cartilage. In the moxibustion group, cartilage surfaces of the femoral condyle and tibial plateau were regular and covered by a hyaline cartilage layer. Although inflammatory proliferation and fibrosis were still present, they were significantly reduced compared with those in the model group.

In the normal group, the cartilage tissue exhibited a smooth surface and intact structure. The chondrocytes were arranged in an orderly manner, with marginal cells being oblate and isolated, whereas deep chondrocytes appeared round or oval. Homologous cell groups were observed, with centrally located nuclei and uniform, full cytoplasm. The tide line was distinct. In the model group, the surface layer of the cartilage tissue was indistinct; a large number of proliferative tissues covered the surface. The layered structure of the cartilage was disordered, the tide line was blurred, fibrosis-like changes in the cartilage matrix were significant, the number of chondrocytes was reduced, and pyknosis and dissolution of the nuclei were obvious. In the moxibustion group, cartilage damage was mitigated, with a less smooth surface and fibrosis-like changes. Proliferative tissue was notably reduced, the chondrocyte layer remained visible, and the tide line appeared irregular. Some areas exhibited clustered chondrocyte proliferation, whereas others exhibited nuclear pyknosis and dissolution.

**Figure 3:**
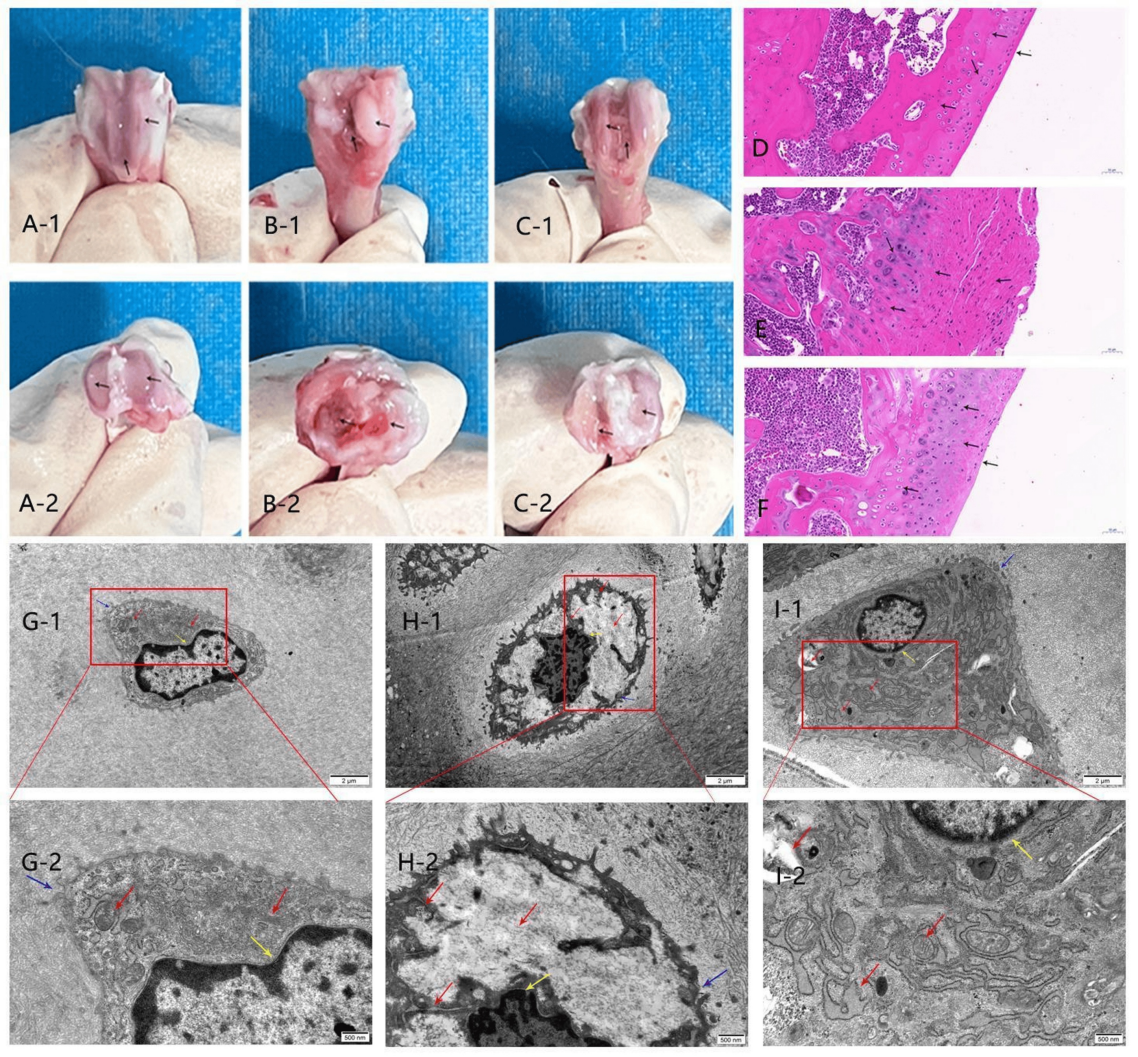
Comparison of histomorphology of rat knee cartilage in each group. A-1 shows the femoral condyle of the knee joint in normal rats, and A-2 shows the tibial plateau of the knee joint in normal rats. B-1 shows the femoral condyle of the knee joint in the model group, and B-2 shows the tibial plateau of the knee joint of rats in the model group. C-1 shows the femoral condyle of the knee joint of rats in the moxibustion group, and C-2 shows the tibial plateau of the knee joint of rats in the moxibustion group. D shows the HE staining observation of knee articular cartilage in the normal group of rats (scale = 50 μm). E shows the HE staining observation of knee articular cartilage in the model group of rats (scale = 50 μm). F shows the HE staining observation of knee articular cartilage in the moxibustion group of rats (scale = 50 μm). G-1/G-2 shows the transmission electron microscope observations of knee articular chondrocytes in the normal group of rats (scale = 2 μm/500 nm). H-1/H-2 shows the transmission electron microscope observations of knee articular chondrocytes in the model group of rats (scale = 2 μm/500 nm). I-1/I-2 shows the moxibustion group. The chondrocytes of the rat knee joint were observed by transmission electron microscopy (scale = 2 μm/500 nm). The black arrow indicates the important observation area of cartilage tissue. Red arrows indicate the state of the cartilage cytoplasm and organelles. The yellow arrow indicates the status of the chondrocyte nucleus. The blue arrow indicates the state of the chondrocyte membrane.

TEM analysis revealed that in the normal group, the chondrocytes exhibited intact membranes, normal microvilli structures, intact nuclear membranes, slightly margined chromatin, abundant rough endoplasmic reticulum, a visible Golgi apparatus, a clear mitochondrial structure, and well-arranged cristae. In contrast, the model group presented condensed chromatin, shrunken nuclear membranes, dissolved cell membranes, broken microvilli scattered in the matrix, dissolved and empty cytoplasm, significantly reduced and broken endoplasmic reticulum, and swollen or vacuolized mitochondria. The moxibustion group maintained intact chondrocyte membranes, partially broken microvilli, reduced nuclear pyknosis, visible endoplasmic reticulum and mitochondria, some dilated endoplasmic reticulum, and significant improvement in cytoplasmic dissolution and degeneration (Figure 3).

**Figure 4:**
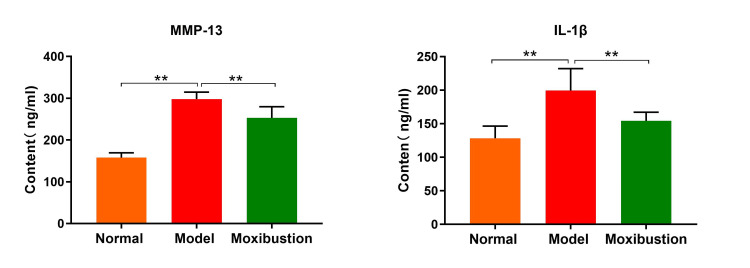
Comparison of matrix metalloproteinase-13 (MMP-13) and interleukin-1β (IL-1β) content in synovial tissue of rats in each group. n=8 per group. Data are expressed as mean ± SD and analyzed using one-way ANOVA followed by Tukey's test. Significance levels are indicated as follows: ** for *P*<0.01.

Compared with those in the normal group, MMP-13 and IL-1β levels in synovial tissue in the model group were significantly greater (P < 0.0001). Compared with those in the model group, MMP-13 and IL-1β levels in synovial tissue in the moxibustion group were significantly lower (P < 0.01) (Figure 4).

**Figure 5:**
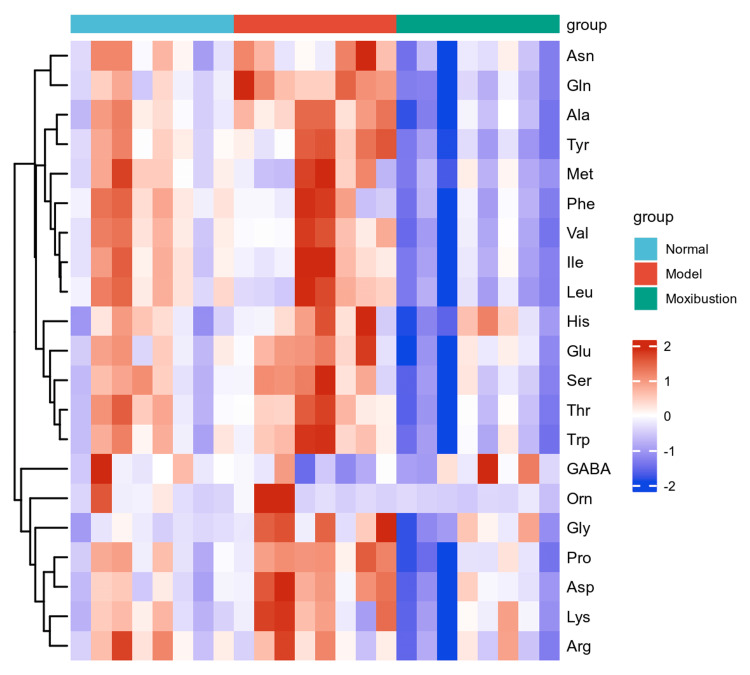
Overall heat map of cluster analysis Columns in the figure represent samples, rows represent metabolites, and the relative contents of metabolites are displayed in different shades of red and blue.

Using LC-MS results, targeted quantification was conducted on the samples, followed by cluster analysis, multivariate statistical analysis, differentially abundant metabolite identification, and metabolic pathway analysis based on the quantitative data. Cluster analysis identified metabolic patterns of metabolites across various experimental conditions. The statistical analysis and visualization of the cluster analysis in this experiment were carried out using the R language. The ComplexHeatmap package was utilized to create a hierarchical cluster heatmap, visualizing the relative quantitative values of metabolites. The relative content in the map is displayed in different colors, with columns representing samples and rows representing metabolites. The study revealed that amino acid levels in the cartilage of rats increased in the model group, whereas the moxibustion group demonstrated significant suppression of amino acid metabolism (Figure 5).

**Figure 6:**
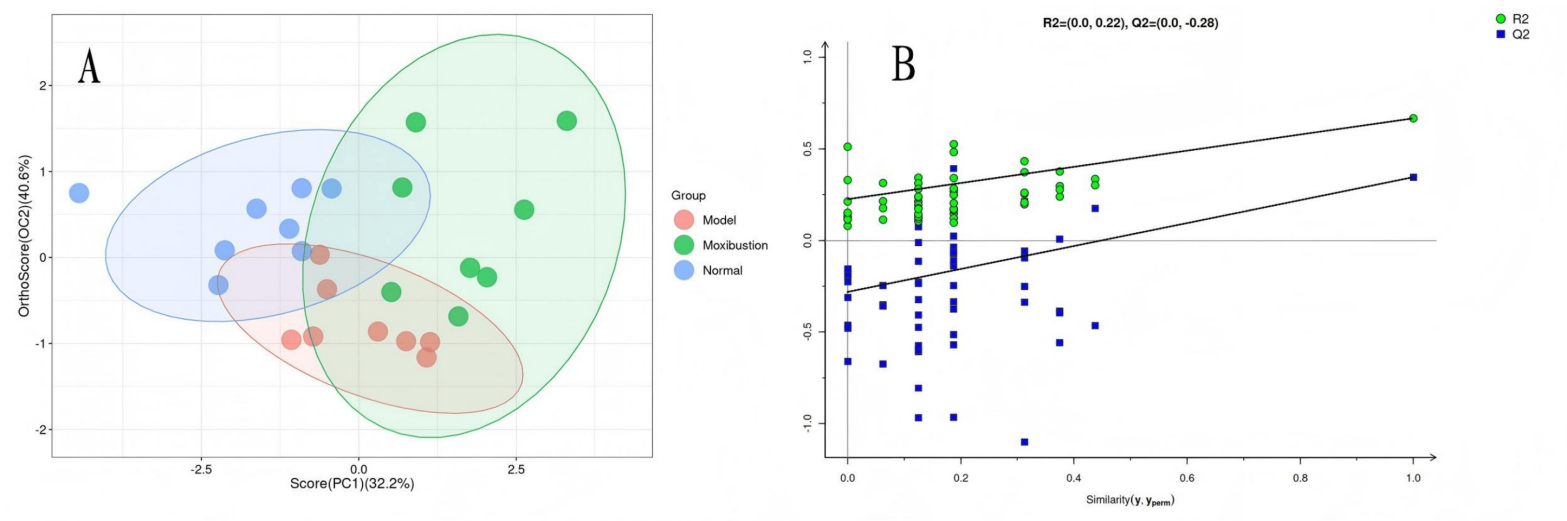
Presents the analysis results from orthogonal projections to latent structures discriminant analysis (OPLS-DA). A is the OPLS-DA score chart, and B is the OPLS-DA model permutation test chart.

Metabonomic data are multidimensional and highly correlated among some variables. Traditional univariate analysis is limited in its ability to efficiently and comprehensively extract potential information from data. Accordingly, using multivariate statistical methods such as orthogonal projections to latent structures discriminant analysis (OPLS-DA) is essential for dimensionality reduction and classification of metabonomic data. In this experiment, the data were first standardized and log2-transformed. Visual analyses using OPLS-DA were conducted with the R language ropls package, which also automatically performs cross-validation and permutation tests. A 7-fold cross-validation was employed to compute the Q2 values in the development of OPLS-DA models. The final OPLS-DA model underwent a permutation test with 200 iterations, where the classification variable y was randomly reordered to derive R2 and Q2 values for the random models, resulting in a permutation test plot. In OPLS-DA, the sample groups demonstrated a good separation trend (Figure 6A), and the evaluation parameters of the model were R2 > 0.5 and Q2 > 0.3 (Table 1). After the permutation test, Q2 < 0 (Figure 6B), indicating that the discrimination and prediction rates of the model were stable and reliable and that the experimental results were credible.

**Table 1:** Evaluation parameters of orthogonal projections to latent structures discriminant analysis (OPLS-DA) model. Pre is the primary component, where R2X denotes the interpretability of X variables, R2Y indicates the interpretability of Y variables, and Q2 signifies the model's predictability. Model stability increases as R2Y and Q2 values approach 1. The model's stability is relatively stable when Q2 > 0.3. The maximum values of R2 and Q2 are 1.

Pre	R2X(cum)	R2Y(cum)	Q2(cum)
1+4+0	0.73	0.67	0.35

**Figure 7:**
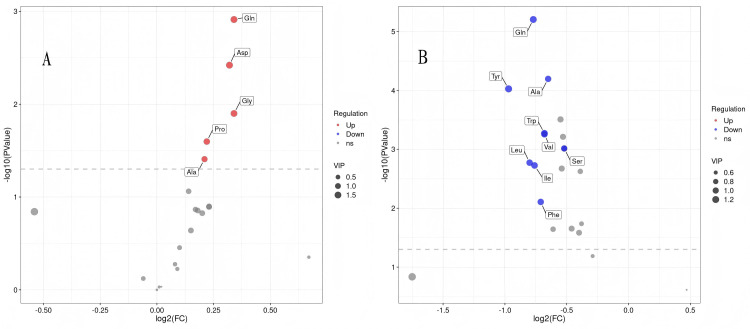
Volcano plots of differential metabolites. A is the comparison between the model group and the normal group, while B is the comparison between the moxibustion group and the model group.The abscissa in the plot represents the log2 transformation of the fold change (FC) value, and the ordinate represents the -log10 transformation of the *P* value.The red dot represents the up regulation of differential metabolites, the blue dot represents the down regulation of differential metabolites, and the gray dot represents the metabolites that have not passed the screening of differential parameters.

Statistical analysis of metabolite differences between the two groups was conducted via FC and t tests, which yielded FC values and P values indicating statistical significance. The OPLS-DA model's variable importance in projection (VIP) was utilized to aid in identifying differentially abundant metabolites. Differentially abundant metabolites were identified based on the criteria of FC > 1, VIP > 1, and a t-test P < 0.05, and a volcano plot illustrating these metabolites between groups was created (Figure 7). The plot reflects the distribution of metabolites. The model group presented significantly higher levels of glutamine, alanine, proline, glycine, and aspartic acid than did the normal group (P < 0.05). Compared with those in the model group, the concentrations of glutamine, alanine, tyrosine, tryptophan, valine, serine, isoleucine, leucine, and phenylalanine in the moxibustion group were significantly lower (P < 0.05).

**Figure 8:**
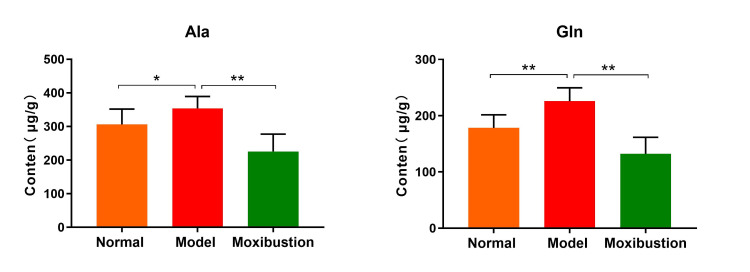
Comparison of the content of the final screened differential amino acids in the knee joint cartilage of rats among each group. n=8 per group. Data are expressed as mean ± SD and analyzed using an unpaired t-test. Significance levels are indicated as follows: * for *P*<0.05 and ** for *P*<0.01. Ala: alanine; Gln: glutamine

**Figure 9:**
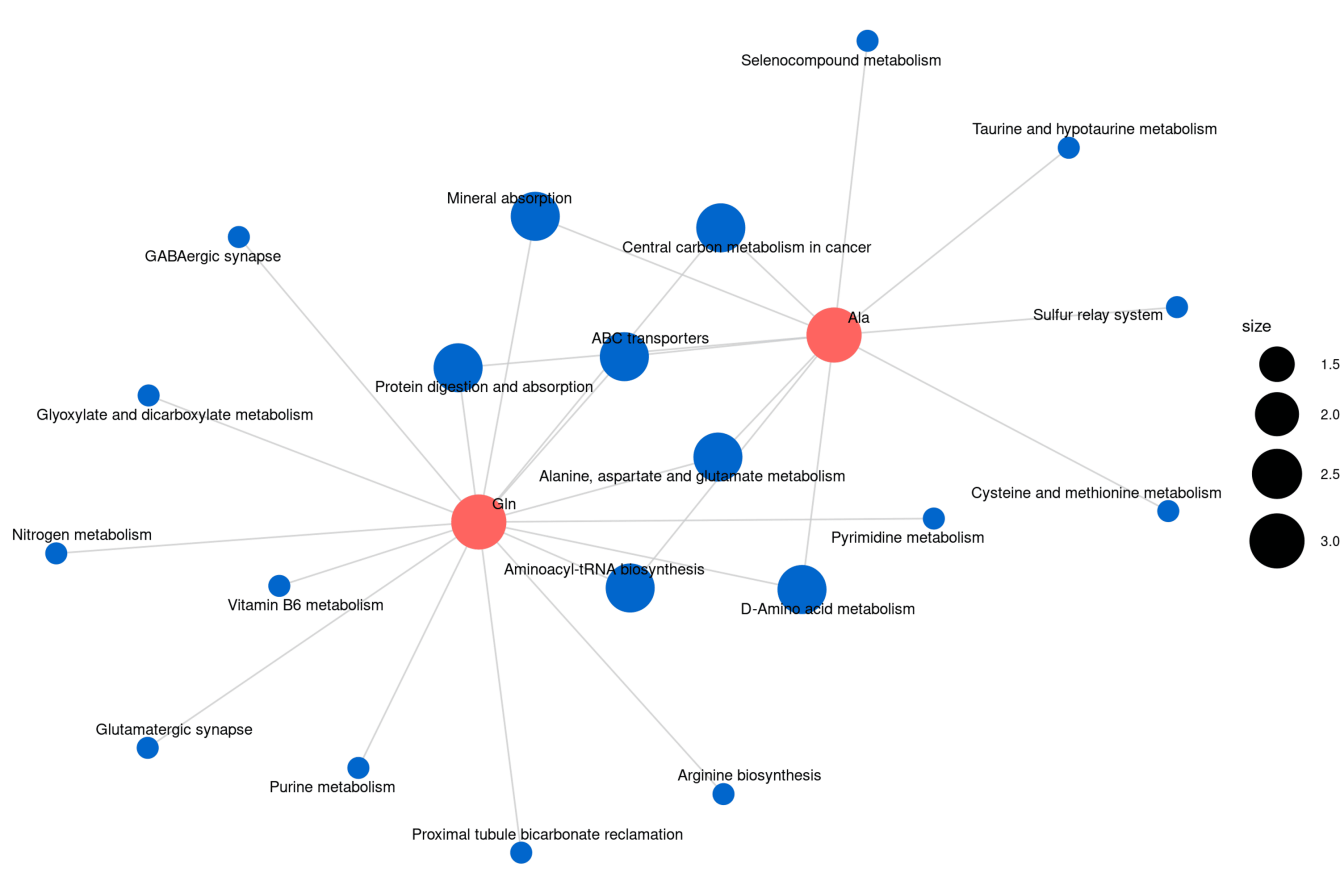
Kyoto Encyclopedia of Genes and Genomes (KEGG) metabolic pathway network of glutamine and alanine. The blue dot indicates the metabolite enrichment pathway, the red dot indicates the target amino acid, and the size of the blue dot indicates the number of amino acids associated with it.

**Figure 10:**
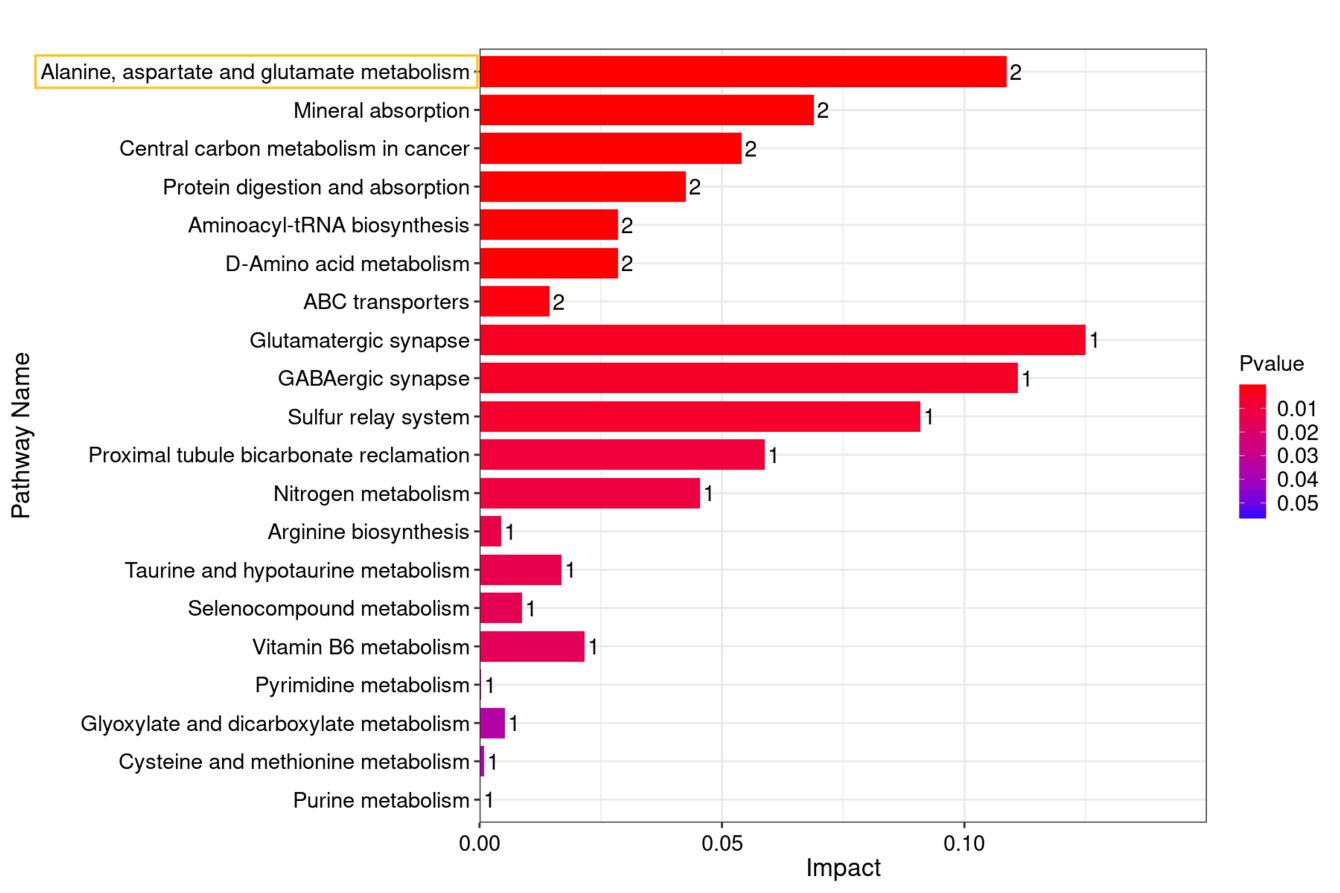
Bar graph of Kyoto Encyclopedia of Genes and Genomes (KEGG) pathway enrichment analysis for glutamine and alanine. The x-axis denotes the influence value, while the y-axis indicates the names of the enriched metabolic pathways.

**Figure 11:**
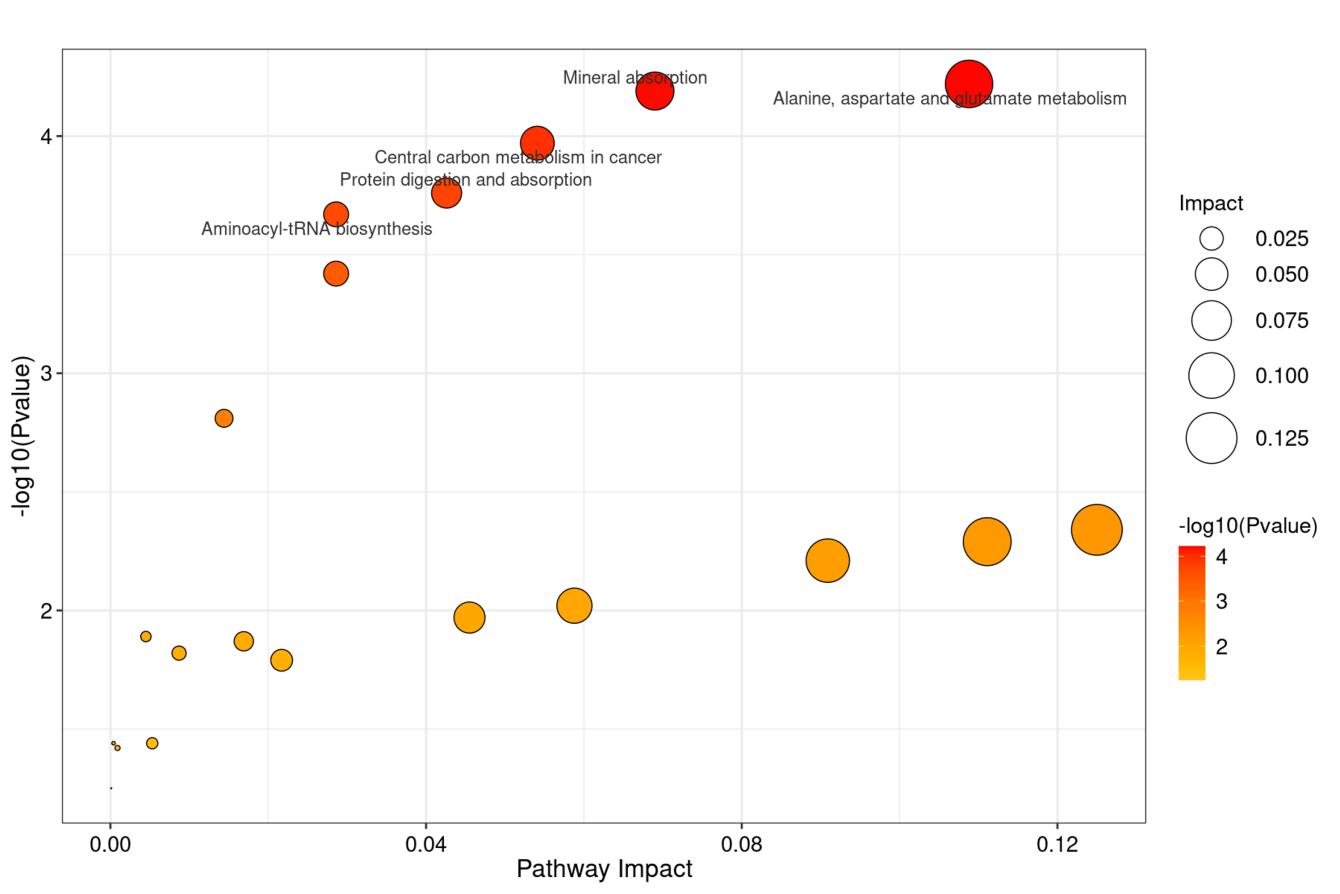
Presents a scatter plot illustrating the Kyoto Encyclopedia of Genes and Genomes (KEGG) pathway enrichment of differential metabolites. The x-axis denotes the KEGG enrichment degree, while the y-axis indicates the p-value from the hypergeometric distribution test, highlighting the relationship between the KEGG enrichment influence value and the p-value.

On this basis, the amino acid metabolites that showed statistically significant differences in all inter-group comparisons were ultimately identified as glutamine and alanine (Figure 8). A Kyoto Encyclopedia of Genes and Genomes metabolic pathway enrichment analysis was performed using the two differentially abundant metabolites, resulting in a network diagram of the differential metabolic pathways (Figure 9). Metabolic pathways exhibiting significant differences (P < 0.05) were identified (Figures 10, 11). The findings indicated that the metabolic pathways involving alanine, aspartate, and glutamate were the most important among the amino acid pathways.

## Discussion

KOA belongs to the categories of 'bone arthralgia' and 'tendon arthralgia' in TCM. Moxibustion has been used to treat knee joint diseases since ancient times. ST36 is a classic health-maintenance acupoint valued by TCM physicians throughout history, and is also a primary acupuncture and moxibustion point used in the management of KOA. Clinical studies have demonstrated that moxibustion significantly relieves KOA symptoms, improves joint function, and enhances the quality of life of the patient. Its therapeutic mechanisms are related to inhibition of inflammatory responses, promoting joint tissue repair, and modulation of immune and intestinal flora functions [13]. Normal articular cartilage lacks both vessels and nerves and therefore does not generate pain. However, the imbalance of cartilage matrix metabolism and abnormal mechanical load leads to cartilage erosion and injury, causing secondary inflammatory reactions in surrounding tissues. The stimulation of angiogenesis and sensory nerve invasion into previously non-innervated areas is an important reason for the induction of pain. The increased activity of matrix metalloproteinases promotes the degradation of the extracellular matrix of cartilage, damages cartilage, and releases pro-inflammatory cytokines, which induce pain sensitization in surrounding tissues and self-circulate inflammation, resulting in pain and joint damage [14,15]. The results revealed that joint swelling in the KOA model rats was obvious, the pain threshold value was significantly decreased, the articular cartilage surface exhibited marked inflammatory tissue proliferation and erosion, and the cartilage tissue and chondrocytes presented obvious pathological damage. In addition, the contents of MMP-13 and IL-1β in synovial tissue increased. After moxibustion intervention, the above indicators significantly improved, suggesting that moxibustion can effectively reduce KOA synovial inflammatory reactions, improve cartilage damage, and relieve clinical symptoms.

In KOA pathogenesis, amino acid metabolites influence the release of inflammatory mediators and neuronal excitability and pain perception, inflammatory signaling, and cellular energy metabolism. Amino acid metabolism is closely associated with cartilage matrix proteins, and cartilage collagen degradation leads to the release of large amounts of amino acids. Previous studies have demonstrated that amino acids and their derivatives play important roles in cartilage injury, synovitis, and chronic pain in KOA patients and may be used as potential biomarkers to predict KOA [16]. Previous studies have also revealed that the amino acid metabolic pathways in the joint microenvironment of KOA patients change significantly, with the levels of aspartic acid, proline, arginine, and phenylalanine in synovial fluid increasing significantly. The metabolism of alanine, aspartic acid, glutamate, arginine, and proline in serum also increases significantly [17,18]. The results of LC-MS-targeted amino acid metabonomics analysis in this study were consistent with these findings. In KOA model rats, the cartilage levels of glutamine, alanine, proline, glycine, and aspartic acid are significantly elevated. Moxibustion notably decreased glutamine and alanine levels, indicating its potential anti-inflammatory, analgesic, and cartilage-protective effects through the modulation of amino acid metabolism, particularly alanine, aspartic acid, and glutamate, in the KOA cartilage microenvironment.

Under conditions of local inflammation and hypoxia in KOA, the energy demand of joint tissue increases rapidly, and the cells accelerate the production of ATP through anaerobic glycolysis, resulting in a significant increase in pyruvate production. Excess pyruvate is catalyzed by lactate dehydrogenase to convert it to lactic acid, which causes the production and accumulation of lactic acid and aggravates joint inflammation and cell death. Alanine can be converted into glutamic acid and pyruvate through the alanine-glucose cycle and can participate in the regulation of energy metabolism [19]. Research indicates that increased levels of alanine, pyruvate, and lactic acid are associated with internal environment disorders such as hypoxia and acidosis in KOA joints, contributing to muscle soreness and pain [20]. Additionally, elevated alanine levels are linked to subchondral bone sclerosis in KOA, serving as a pathological marker of impaired mitochondrial function in chondrocytes and joint degradation [21].

This study revealed a significant increase in alanine content in the cartilage of KOA model rats, suggesting a compensatory energy demand in damaged chondrocytes that may exacerbate local joint inflammation and cartilage damage. Moxibustion can effectively improve this condition. In addition, studies have demonstrated that chondrocytes in the inflammatory state of KOA rely on glutamine as a source of energy and that the expression of various glutamine metabolic enzymes in cartilage is increased. Glutamine deprivation triggers metabolic reprogramming and inhibits glycolysis and NF-κB inflammatory signaling pathway activity, thereby weakening the inflammatory response [22]. Our experimental results revealed that the glutamine content in the cartilage tissue of KOA model rats increased significantly, and that inhibiting glutamine metabolism is one of the important mechanisms by which moxibustion reduces the local inflammatory response.

This study investigated the effects of moxibustion intervention on knee joint swelling, the mechanical pain threshold, chondrocyte morphology, and synovial MMP-13 and IL-1β contents in KOA model rats; verified the efficacy of moxibustion in the treatment of KOA from the perspectives of behavior, morphology, and molecular biology; and analyzed the changes in amino acid metabolites in cartilage tissue through targeted metabonomics. Furthermore, moxibustion can regulate the immune response and disordered cartilage synthesis and degradation in KOA, exert anti-inflammatory, analgesic, and cartilage-protective effects by inhibiting the overactive amino acid metabolic pathway in cartilage, and preliminarily elucidate the material basis of the moxibustion effect from the perspective of amino acid metabolism.

However, the study has several limitations. Although we observed significant effects of moxibustion on amino acid metabolites in KOA cartilage and identified several differentially abundant pathways, there are certain differences in pathological changes between chemically induced osteoarthritis and "natural" osteoarthritis; the specific mechanistic relationships among these pathways and the therapeutic outcomes require further experimental validation. Moreover, the roles of individual amino acid metabolites in mediating the effect of moxibustion should be explored using clinical samples and mechanistic studies. Additionally, this study did not include alternative acupoints as controls, making it difficult to confirm whether the therapeutic effects were specific to stimulation at ST36. Future studies should refine the experimental design by incorporating acupoint controls and larger sample sizes to more comprehensively understand the mechanisms underlying the benefits of moxibustion in KOA.

## Conclusions

Moxibustion at ST36 can significantly improve joint swelling, pain, synovitis, and chondrocyte injury in KOA model rats, and regulate the KOA immune response as well as cartilage synthesis and degradation through alanine, aspartate, and glutamate amino acid metabolic pathways, thereby exerting anti-inflammatory, analgesic, and protective effects on cartilage injury.
